# 

**Published:** 1907-01

**Authors:** 


					﻿Vol. XXII.
JANUARY^ 1907.
No.-7.
TEXAS
I' ■ BWSB5' ‘ ffVTVIf ’. .
JOURNAL
“RED BACK”
.. WBLISHED AT AUSTIN, TEXAS, BY
;X;X^>ANIEL, M. D.,	-	“ Proprietor
PRINTED BY THE VON BOECKM ANN-JONES CO.
Subscription, fl .00 a Year, in Advance. -	-	- Single Copies, 10 Cents
Entered at the Postofflce at Austin, Texas, as Second-class Mail Matter
				

